# Predictive Factors of Superior Mediastinal Nodal Metastasis from Papillary Thyroid Carcinoma—A Prospective Observational Study

**DOI:** 10.1371/journal.pone.0148420

**Published:** 2016-02-05

**Authors:** Joo Hyun Woo, Ki Nam Park, Jae Yong Lee, Seung Won Lee

**Affiliations:** 1 Department of Otorhinolaryngology—Head and Neck Surgery, Gachon University, Graduate School of Medicine, Gil Medical Center, Incheon, Korea; 2 Department of Otorhinolaryngology—Head and Neck Surgery, Soonchunhyang University School of Medicine, Bucheon Hospital, Bucheon, Korea; University of Toronto, CANADA

## Abstract

**Objectives:**

The purpose of this study was to demonstrate the incidence rates and predictive factors of superior mediastinal lymph node (SMLN) metastasis in PTC (papillary thyroid carcinoma) patients.

**Methods:**

A prospective observational study was performed between January 2009 and January 2011. PTC patients who had tumors with a maximal diameter greater than 1 cm and clinically negative SMLNs were included in this study. Finally, a total of 217 patients who underwent total thyroidectomy with central compartment neck dissection (CND) and elective superior mediastinal lymph node dissection (SMLND), with or without modified radical neck dissection (MRND) and revisional CND, were included.

**Results:**

Occult SMLN metastasis was present in 15.7% (34/217). Cytological classifications of tumor, *BRAFV600E* mutation, Tumor size, T-stage, perithyroidal extension, lymphovascular invasion, multifocality, and paratracheal pN(+) were not predictive of SMLN metastasis (P > .05), while revision surgery, pretracheal pN(+), and multiple lateral pN(+) were associated with SMLN metastasis. There were no major complications related to SMLND. Transient and permanent hypoparathyroidism was observed in 69 cases (31.8%) and 8 cases (3.6%), respectively.

**Conclusions:**

Despite clinically negative SMLN in preoperative evaluation, SMLN metastasis can be predicted for patients with a PTC tumor size larger than 1 cm, pretracheal LN metastasis, multiple lateral metastasis, and revisional surgery.

## Introduction

Superior mediastinal lymph nodes (SMLNs) at level VI are bordered at only by an imaginary line, not by an anatomical barrier [1,2]. It is suspected that, when metastasis presents in neighboring pretracheal or paratracheal lymph nodes (LNs), the possibility of SMLN metastasis might increase in papillary thyroid carcinoma (PTC) patients. Recurrence in SMLNs can be life-threatening owing to their proximity to vital structures (e.g., the esophagus, trachea, lung, innominate artery, and aortic arch)[3,4]. Risk factors of SMLN metastasis as identified in previous studies are contralateral lateral neck LN metastasis, central lymph node (CLN) metastasis, extensive CLN metastasis, contralateral CLN metastasis, direct extension to SMLN, grossly perithyroidal extension (PTE), medullary or poorly differentiated carcinoma, distant metastasis, and Hurtle cell carcinoma[5–10]. However, no prospective studies have assessed the accuracy of histopathologic information and risk factors for SMLN metastasis in PTC patients. This is the first prospective study to present an analysis of predictive factors for SMLN metastasis. The aim of this study was to investigate the incidence and predictive factors for the presence of occult superior mediastinal LN metastasis and to evaluate the role of elective transcervical SMLND in clinical lymph node-negative patients with a larger than 1 cm PTC.

## Materials and Methods

### Study Population

This observational study was designed prospectively. Patients with PTC underwent thyroid surgery between January 2009 and January 2011 and were enrolled prospectively. PTC was diagnosed by preoperative fine needle aspiration cytology (FNAC), *BRAFV600E* mutation, or intraoperative frozen sections. Among these patients with PTC, those who had PTC with a maximal diameter greater than 1 cm and clinically negative SMLN and were treated with total thyroidectomy with CND (central compartment neck dissection) and elective SMLND (superior mediastinal lymph node dissection) ± MRND (modified radical neck dissection) were included in this study. Patients who had received revisional central neck dissection for regional recurrence at the level VI compartment were also included. Patient treatment was the standard of care. Patients whose pathologic report revealed papillary thyroid microcarcinoma, follicular type, undifferentiated type, obscure sorting of lymph nodes, and the absence of superior mediastinal lymph nodes were excluded. A clinically characterized node-negative superior mediastinum was defined as lacking suspicious metastatic LN on preoperative imaging studies, such as high-resolution ultrasonography and computed tomography. Consequently, a total of 217 consecutive PTC patients were enrolled in this study. There were 181 women and 37 men, ranging in age from 12 to 81 years, with a mean age of 48.7 (SD 13.1) years. All of the patients provided informed consent for their treatment and for their inclusion in this study. This study was approved by the Institutional Review Board of Soonchunhyang University Bucheon Hospital (SCHBC_IRB_09_60). Participants or parents of young participants provided their written informed consents to participate in this study.

### Surgical procedure of SMLND

**Fig 1:**
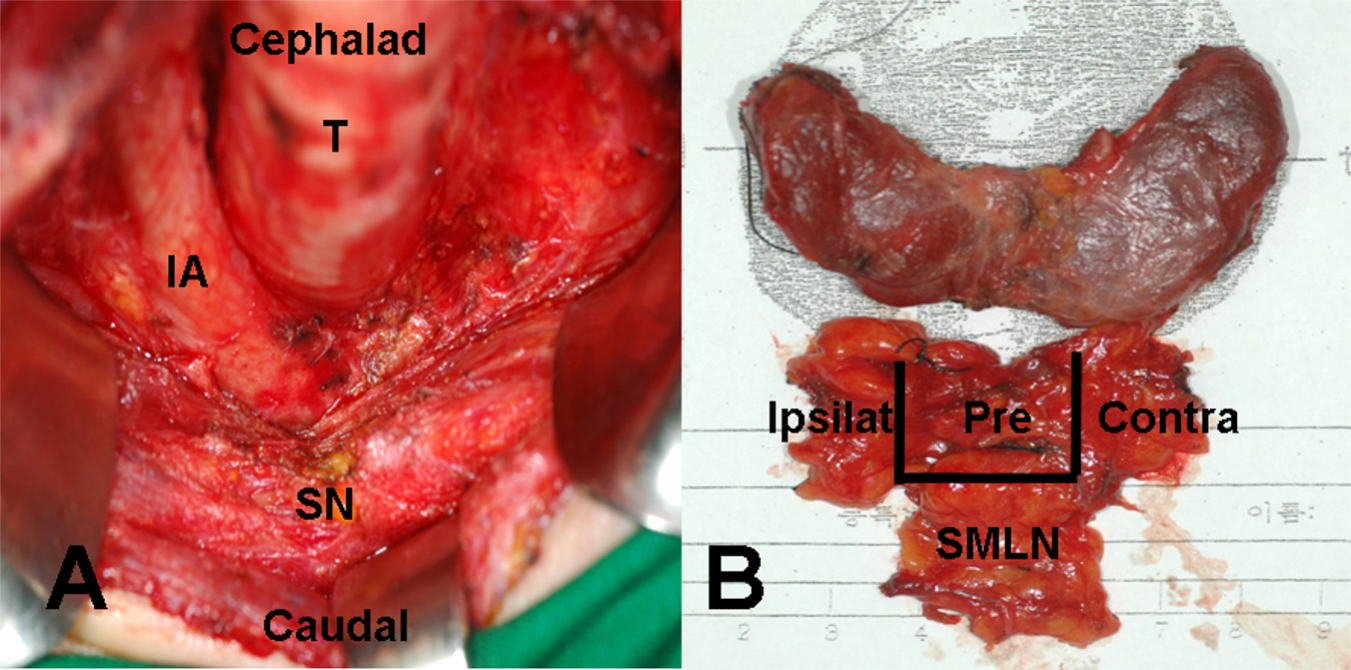
Surgical field and specimen. (A) Total thyroidectomy with central lymph node dissection and superior mediastinal lymph node dissection were performed. (B) The specimen showed thyroid gland and adjacent fibrofatty tissue, including pretracheal, bilateral paratracheal, and superior mediastinal lymph nodes. T: trachea, IA: innominate artery, SN: sternal notch, Pre: pretracheal lymph nodes, Ipsi: ipsilateral paratracheal lymph node, Contra: contralateral paratracheal lymph node, SMLN: superior mediastinal lymph node.

All surgical procedures were performed using the no tie-harmonic scalpel (Johnson & Johnson Ethicon, NJ, USA) thyroidectomy technique by an experienced surgeon. The extent of CND was inferiorly to the sternal notch, laterally to the carotid sheaths, and dorsally to the prevertebral fsascia. SMLND was performed via the transcervical approach without sternotomy and was bordered superiorly to the sternal notch, inferiorly to the left brachiocephalic vein and innominate artery (Fig 1A).

Boundaries of CND and SMLN were labeled with No-2 black silk during the operation, before LN removal. Central lymph nodes (CLNs) are classified as pretracheal, ipsilateral paratracheal, and contralateral paratracheal LN (Fig 1B). Ipsilateral paratracheal LNs were defined as paratracheal lymph nodes located on the same side as the primary thyroid carcinoma. The labeled specimens were sent to the pathology department for permanent section analysis. The total operative time, CND and SMLND time, and blood loss amount were also evaluated during the operation. Complications, such as recurrent laryngeal nerve paralysis, hypoparathyroidism, major vessel tearing, and trachea and esophageal injury, were also checked.

### Evaluation of Postoperative Complications

All patients were clinically evaluated for signs and symptoms of hypocalcemia after surgery. Hypoparathyroidism was classified as either temporary or permanent: temporary hypoparathyroidism was defined as a parathyroid hormone (PTH) level that could not recover above 10 IU/ml until postoperative 1 month or the need for calcium due to persistent hypocalcemia symptoms; permanent hypoparathyroidism was defined as a PTH level that could not reach above 10 IU/ml until postoperative 1 year or persistent hypocalcemia symptoms. Patients who developed hypocalcemia were given oral calcium with active vitamin D replacement. Follow-up data on parathyroid function (serum ionized, total calcium, and intact PTH) were obtained.

The mobility of the vocal fold was examined after surgery at each follow-up using a flexible fiberscope to check for recurrent laryngeal nerve injury.

### Statistical Analysis

Statistical analysis was performed using the SPSS version 11 software. Univariate and multivariate analyses of the relationships between SMLN metastasis and clinico-pathologic characteristics of the primary lesion or lymph node metastasis were performed using the Fisher exact test and binary logistic regression. A *P* value less than 0.05 was considered to be statistically significant.

## Results

### Operative Examination

Among the 217 patients who were enrolled in this study, 194 patients underwent total thyroidectomy with CND and SMLND, and 37 of the 194 underwent MRND. Twenty-three of the 217 underwent revisional CND (Table 1).

**Table 1:** Demographic information. TT; total thyroidectomy, CND; central compartment neck dissection, SMLND; superior mediastinal lymph node dissection, MRND; modified radical neck dissection.

**Procedure**	**N (%)**
TT+CND+SMLND	157 (72.4)
TT+CND+SMLND+MRND	37 (17.1)
Revision CND	23 (10.6)
Total	217 (100)
**Time of procedure**	**Minutes (SD / Range)**
Total operation time (TT+CND+SMLND±MRND)	109.8 (69.1 / range 40–780)
CND+SMLND	18.2 (4.3 / 10–30)

The mean operative time for CND including SMLND was 18.2 minutes; the total operation time, including total thyroidectomy, CND, and SMLND with or without lateral neck dissection, was 109.8 minutes. The mean amount of blood loss during the operation was 23.7 ml (Table 1).

### Pathologic Examination

The mean size of the primary thyroid tumor was 16.3 mm (SD 0.9), and the mean T stage of the primary thyroid tumor was 2.53 (SD 0.4). Lymphovascular extension, macroscopic perithyroidal extension, and multifocality of the primary tumor were found in 66.8%, 6.9%, and 22.6%, respectively (Table 2).

**Table 2:** Pathologic characteristics of thyroid carcinoma and lymph node metastasis.

**Characteristics of the thyroid tumor**	**Mean / SD / Range**
Tumor size (mm)	16.3 / 0.9 / 1.1–6.0
T-stage	2.53 / 0.4 / I–IV
	**N (%)**
Lymphovascular extension	145 (66.8)
Macroscopic perithyroidal extension	15(6.9)
Multifocality of the primary tumor	49 (22.6)
**Characteristics of lymph nodes**	**Mean (SD)**
Number of superior mediastinal pN(+)	1.5 (2.1)
Number of retrieved central lymph nodes	8.4 (6.6)
Number of central pN(+)	1.7 (1.8)
	**N (%)**
Superior mediastinal pN(+)	34 (15.7)
Ipsilateral paratracheal pN(+)	66 (30.4)
Pretracheal pN(+)	70 (32.3)
Contralateral paratracheal pN(+)	40 (18.4)
Multiple central pN(+)	50 (23.0)
Lateral neck pN(+) in 17.1%	37 (17.1)

Thirty-four (15.7%) of 217 patients had pathologic SMLN metastasis, and the mean number of positive SMLNs was 1.5 (SD 2.1). The mean total number of retrieved CLNs was 8.4 (SD 6.6), and the mean number of positive CLNs was 1.7 (SD 1.8). Ipsilateral paratracheal LN metastasis (pathologic positive node, pN(+)) was detected in 30.4%, pretracheal pN(+) in 32.3%, contralateral paratracheal pN(+) in 18.4%, multiple central pN(+) in 23.0%, and lateral neck pN(+) in 17.1% of the patients (Table 2).

The relationship between SMLN metastasis and clinico-pathologic factors in the 217 patients was analyzed. In univariate analysis, the rate of SMLN metastasis was significantly higher in revision surgery and in the presence of contralateral paratracheal pN(+), pretracheal pN(+), and multiple central pN(+) (P < 0.05). There were no statistically significant differences in the rates of SMLN metastasis among Bethesda classification of FNAC, *BRAFV600E* mutation, the size of tumor, T-stage, macroscopic perithyroidal extension, lymphovascular invasion, multifocality of the primary tumor, and ipsilateral paratracheal pN(+) (Table 3). Multivariate analysis was performed for factors that were shown to be significant in the univariate analysis. In the multivariate analysis, the rates of SMLN metastasis were significantly higher in revision surgery (P = 0.011, odd ratio 5.290) and in the presence of pretracheal pN(+) (P = 0.045, odd ratio 2.899) and multiple lateral neck pN(+) (P = 0.026, odds ratio 3.644) (Table 4).

**Table 3:** Clinico-pathologic factors related to SMLN metastasis in 217 papillary thyroid carcinoma patients: univariate analysis. Ipsi; ipsilateral, Contra; contralateral, SMLN: superior mediastinal lymph node, Small tumor: ≤ 2 cm, large tumor: > 2 cm, Early T stage: TI and TII, Advanced T stage: TIII and TIV, PTE: perithyroidal extension excluding microscopic PTE,

Clinico-patholgic factors		No. of patients with	*P* value
		a positive SMLN (%)	
Bethesda classification	I—IV	7/71 (9.9)	0.148
	V	21/102 (20.6)	
	VI	6/44 (13.6)	
*BRAFV600E* mutation	Yes	14/87 (16.1)	0.966
	No	5/30 (16.7)	
Size of tumor	< 2 cm	25/184 (13.5)	0.286
	≥2 cm	7/33 (21.2)	
T stage	Early	5/59 (8.5)	0.093
	Advanced	29/158 (18.4)	
Perithyroidal extension	Yes	26/146 (17.8)	0.159
	No	7/71 (9.9)	
Lymphovascular Invasion	Yes	3/15 (20.0)	0.710
	No	31/202 (15.3)	
Multifocality of the primary	Yes	10/50 (20.0)	0.257
tumor	No	22/167 (13.2)	
Revision surgery	Yes	12/23 (52.2)	0.000^*^
	No	22/194 (11.3)	
Ipsi-paratracheal pN(+)	Yes	15/69 (21.7)	0.063
	No	17/148 (11.4)	
Contra-paratracheal pN(+)	Yes	14/54 (25.9)	0.007^*^
	No	17/163 (15.4)	
Pretracheal pN(+)	Yes	22/70 (31.4)	0.000^*^
	No	12/147 (8.2)	
Multiple central pN(+)	Yes	15/50 (30.0)	0.003^*^
	No	19/167 (11.4)	
Multiple lateral neck pN(+)	Yes	17/37 (30.0)	0.000^*^
	No	17/180 (9.4)	

*: *P* < 0.05 in *Chi* squared test.

**Table 4:** Multivariate logistic regression for SMLN metastasis. Contra: contralateral, SMLN: superior mediastinal lymph node, SE: standard error, Exp (ß): exponential (ß), CI: confidence interval,

Variables	ß (SE)	*P* value	Exp (ß)	95% CI	
				Lower	Upper
Revision surgery	1.666 (0.659)	0.011^*^	5.290	1.454	19.250
Contra-paratracheal pN(+)	0.834 (0.561)	0.137	2.302	0.766	6.918
Pretracheal pN(+)	1.293 (0.580)	0.026^*^	3.644	1.169	11.360
Multiple central pN(+)	0.119 (0.652)	0.855	1.127	0.314	4.047
Multiple lateral neck pN(+)	1.064 (0.532)	0.045^*^	2.899	1.022	8.222

*: *P* < 0.05 in logistic regression.

### Complications Related to Surgery

There were no major adverse effects related to SMLND, such as major vessel injury, pneumothorax, recurrent laryngeal nerve injury, and tracheal or esophageal injury. Transient hypoparathyroidism developed in 69 cases (31.8%), while permanent hypoparathyroidism was identified in 8 cases (3.6%). Tearing of the brachiocephalic vein occurred in 2 cases (0.9%) during SMLND, but this was easily controlled by simple electrocauterization or suture ligation.

## Discussion

This study aimed to identify predictive factors of elective SMLN metastasis and to clarify the surgical indications that SMLND is necessary. SMLNs are categorized as level VI LNs by an imaginary line, not an anatomical barrier. SMLNs are directly connected to the lymphatic chain of the central compartment, and metastatic PTC could pass through the arbitrary limit between these regions[2]. The suggested mechanism of SMLN metastasis of thyroid carcinoma is downward lymphatic drainage from CND by gravity and direct extension of the thyroid tumor into the superior mediastinum[1,11–13]. In many patients, lymph node metastases in the central compartment do not appear abnormal preoperatively with imaging or by inspection at the time of surgery[14,15]. Untreated subclinical lymph node metastasis could increase regional lymph node recurrence, and this is unlikely to be eradicated by radioactive iodine therapy[16]. Superior mediastinal recurrence may increase the complexity of surgical treatment procedures and induce fatal problems due to its close proximity to the lung apex and innominate artery[17]. It is necessary to identify risk factors for SMLN metastasis in PTC patients to reduce fatal regional recurrence by performing elective SMLND during the primitive operation. However, previous studies were retrospective, only included heterogeneous patient groups, and showed highly variable incidence rates of SMLN metastasis from 5.6% to 100%[17,18]. To our knowledge, this study is the first prospective study concerning the predictive factors of SMLN metastasis in PTC patients. Our study included a control patient group whose members had thyroid carcinoma with a maximal diameter greater than 1 cm and clinically negative SMLN. For these reasons, our study showed reliable results for SMLN metastasis in papillary thyroid carcinoma. Although previous studies have reported that tumor size, lymphovascular invasion, and perithyroidal extension were predictive factors of lymph node metastasis in PTC, particularly tumor size because it is an independent risk factor for central LN metastasis[19,20], this study revealed that these factors did not show significant relationships with SMLN metastasis. This study showed a 15.7% incidence of SMLN metastasis, possibly indicating that elective transcervical SMLND was not routinely recommended. However, pretracheal LN metastasis, multiple lateral neck metastasis, and revision surgery were highly associated with SMLN metastasis in our study, and these could be considered significant predictive factors of SMLN metastasis.

Surgeons prefer to avoid SMLND because of the incidence of surgical morbidity related to the procedure, stemming from major vessel injury, hypoparathyroidism, recurrent laryngeal nerve injury, pneumothorax, and pleural effusion[21]. However, a previous study has already reported the safety of SMLND without life-threatening complications[22]. Moreover, transcervical SMLND is a useful and safe surgical approach to access the superior mediastinum between the sternal notch and the innominate vasculature[1,22]. However, it is worth noting that one study showed a high rate of transient hypoparathyroidism (70%) and of permanent hyperparathyroidism (50%)[7]. Our study showed acceptable rates of hypoparathyroidism (transient, 31.8%; permanent, 3.6%) with preservation of blood supply to the parathyroid glands[23]. An elective transcervical SMLND, using a no-tie harmonic scalpel technique, is a safe method to remove lymph node groups between the sternal notch and the brachiocephalic (innominate) vasculature.

In summary, the elective transcervical SMLND is not routinely recommended for PTC patients. However, for patients with multiple lateral LN metastasis, pretracheal central LN metastasis, and/or regional recurrence at the level VI compartment, transcervical SMLND could be recommended, although a study of preoperative imaging showed clinically negative SMLNs. Long-term follow-up is necessary to evaluate the impact of SMLNs on locoregional control and disease-free survival for papillary thyroid carcinoma in the future.

## Conclusion

Despite clinically negative SMLNs in preoperative evaluation, SMLN metastasis can be predicted in patients with a larger than 1 cm PTC and pretracheal LN metastasis, multiple lateral neck metastases, and regional recurrence at the level VI compartment.
